# Serum Neurofilament Light is elevated in COVID-19 Positive Adults in the ICU and is associated with Co-Morbid Cardiovascular Disease, Neurological Complications, and Acuity of Illness

**DOI:** 10.26502/fccm.92920221

**Published:** 2021-10-13

**Authors:** Meredith Hay, Lee Ryan, Matthew Huentelman, John Konhilas, Christina Hoyer-Kimura, Thomas G Beach, Geidy E Serrano, Eric M Reiman, Kaj Blennow, Henrik Zetterberg, Sairam Parthasarathy

**Affiliations:** 1 Physiology, University of Arizona, Tucson, AZ, USA; 2 Sarver Heart Center, University of Arizona, Tucson, AZ, USA; 3 Evelyn F. McKnight Brain Institute, University of Arizona, Tucson, USA; 4 Department of Psychology, University of Arizona, Tucson, AZ, USA; 5 Department of Medicine, College of Medicine, University of Arizona, Tucson, Arizona, USA; 6 Division of Pulmonary, Allergy, Critical Care and Sleep Medicine, Department of Medicine, University of Arizona, Tucson, Arizona, USA; 7 UAHS Center for Sleep and Circadian Sciences, University of Arizona, Tucson, Arizona, USA; 8 ProNeurogen, Inc, Tucson, AZ, USA; 9 Neurogenomics Division, TGen, Phoenix, AZ, USA; 10 Banner Alzheimer’s Institute and Arizona Alzheimer’s Consortium, Phoenix, AZ, USA; 11 Laboratory of Neuropathology, Banner Sun Health Research Institute, Sun City, AZ, USA; 12 Clinical Neurochemistry Laboratory, Sahlgrenska University Hospital, Mölndal, Sweden; 13 Institute of Neuroscience and Physiology, The Sahlgrenska Academy at University of Gothenburg, Mölndal, Sweden; 14 Department of Neurodegenerative Disease, UCL Institute of Neurology, London, United Kingdom; 15 UK Dementia Research Institute at UCL, London, United Kingdom

**Keywords:** Serum Neurofilament, SARS-CoV-2, Alzheimer’s disease, Dementia, Co-Morbid Cardiovascular Disease

## Abstract

In critically ill COVID-19 patients, the risk of long-term neurological consequences is just beginning to be appreciated. While recent studies have identified that there is an increase in structural injury to the nervous system in critically ill COVID-19 patients, there is little known about the relationship of COVID-19 neurological damage to the systemic inflammatory diseases also observed in COVID-19 patients. The purpose of this pilot observational study was to examine the relationships between serum neurofilament light protein (NfL, a measure of neuronal injury) and co-morbid cardiovascular disease (CVD) and neurological complications in COVID-19 positive patients admitted to the intensive care unit (ICU). In this observational study of one-hundred patients who were admitted to the ICU in Tucson, Arizona between April and August 2020, 89 were positive for COVID-19 (COVID-pos) and 11 was COVID-negative (COVID-neg). A healthy control group (n=8) was examined for comparison. The primary outcomes and measures were subject demographics, serum NfL, presence and extent of CVD, diabetes, sequential organ failure assessment score (SOFA), presence of neurological complications, and blood chemistry panel data. COVID-pos patients in the ICU had significantly higher mean levels of Nfl (229.6 ± 163 pg/ml) compared to COVID-neg ICU patients (19.3 ± 5.6 pg/ml), Welch’s t-test, p =.01 and healthy controls (12.3 ± 3.1 pg/ml), Welch’s t-test p =.005. Levels of Nfl in COVID-pos ICU patients were significantly higher in patients with concomitant CVD and diabetes (n=35, log Nfl 1.6±.09), and correlated with higher SOFA scores (r=.5, p =.001). These findings suggest that in severe COVID-19 disease, the central neuronal and axonal damage in these patients may be driven, in part, by the level of systemic cardiovascular disease and peripheral inflammation. Understanding the contributions of systemic inflammatory disease to central neurological degeneration in these COVID-19 survivors will be important to the design of interventional therapies to prevent long-term neurological and cognitive dysfunction.

## 1. Introduction

Neurological complications following SARS-CoV-2 infection and been reported by a number of investigators [1–3]. In addition, there is overlap of the risk factors in patients with severe COVID-19 and patients at risk for Alzheimer’s Disease Related Dementias (ADRD) and vascular contributions to cognitive impairment and dementia (VCID). These include age, hypertension, diabetes, cardiac disease, hypercholesterolemia, and pulmonary disease. Recent studies have shown that of ICU admitted COVID-19 patients in France, 84% showed some level of neurological impairment during their hospital admission [4] and there is a high prevalence of neurological involvement in critically ill patients suffering from COVID-19 [5–8]. It has been suggested that SARS-CoV-2 results in damage to the CNS damage via a surge of systemic inflammatory cytokines called Cytokine Storm Syndrome (CSS) [9, 10].

Neurofilament light protein (Nfl) is one of the 3 primary neurofilament isotypes that have been shown to increase in both the cerebrospinal fluid (CSF) and blood in the presence of axonal damage and neurodegeneration [11]. Levels of serum Nfl have been found to be elevated in subjects with a number of neurological degenerative diseases [12–15], as well as those with acute conditions such as hypoxic brain injury [16], cardiac and related surgeries [17, 18], and traumatic brain injury [19]. Recent studies have found increased levels of Nfl in COVID-19 patients [20–22] but the effects of concomitant systemic inflammatory disease such as cardiac disease or diabetes on neuronal injury as measured by NfL in COVID-19 patients has not been previously reported. In the present study we examined the relationship between levels of serum Nfl in COVID-19 positive ICU patients and the presence of cardiovascular disease, diabetes, and acute neurological complications.

## 2. Materials and Methods

### 2.1 Study population

One-hundred patients admitted to the ICU in Tucson, Arizona, USA, were included in this study. Of these, 89 patients were positive for COVID-19 with an average age of 60.8. Of these 89, full clinical data was available on 50 patients. All 50 of the COVID-19 positive (COVID-pos) ICU patients were mechanically ventilated. We compared the COVID-19-pos ICU Nfl data to 11 patients who were admitted to the ICU but were COVID-negative (COVID-neg), with an average age of 65.1. Of these 11, full clinical data were available on 5 patients. Of these five patients, four were mechanically ventilated and one was on a high flow nasal cannula system.

Blood samples for the Nfl assay were obtained on the day of admission to the ICU. The Nfl assay on samples from the ICU patients was performed in the Clinical Neurochemistry Laboratory at the Sahlgrenska University Hospital using the Single molecule array (Simoa) NF-light Advantage and HD-X Analyzer, (Quanterix, Billerica, MA). A single batch of reagents was used; intra-assay coefficients of variation were below 6.8% for all analytes. The Nfl assay on samples from the healthy control cohort were performed by PBL Assay Science, New Jersey, USA, and analyzed using the same Simoa NF-light Advantage kit on an HD-X Analyzer, as per the manufacturer’s instruction (Quanterix, Billerica, MA).

Upon admission, patient’s medical histories and clinical data including primary admission diagnosis, their SOFA score (the SOFA score [23] is derived from scores from six organ systems ranging from 0 (no organ dysfunction) to 4 (severe organ dysfunction) and the individual organ scores are then summed to a total score between 0 and 24), the Glasgow Coma Score (assessed without sedation), Systemic Inflammatory Response Syndrome (SIRS) evaluation, sepsis evaluation, pulmonary function including ventilation rates and volumes, days intubated, renal function, liver function, full chemistry laboratory panel, ICU-related complications, duration of stay in ICU, duration of hospital stay and in-hospital death. Cardiovascular disease (CVD) secondary diagnosis upon admission included the presence of heart failure, hypertension (defined as greater than 140–159/90–99 mm Hg), coronary artery disease, valvular disease, arrhythmia, hyperlipidemia, previous MI, obesity, and smoking history. The CVD score was derived by assigning one point for each of the above cardiovascular related diagnoses and then summing all for the total CVD score. Neurological complications (neurocx) were assessed upon hospital admission and during ICU stay. Levels of ICU delirium were assessed twice daily while in the hospital by trained research personnel using the Richmond Agitation-Sedation Scale (RASS) and Confusion Assessment Method-ICU CAM-ICU [24].

### 2.2 Statistical analysis

Experimental values are expressed as mean ± SE unless otherwise indicated. Comparison of the Nfl levels and log Nfl levels between the 2 ICU groups and the control were analyzed with Welches’t-test with significance determined at a p value < .05. The Welches’t-test was chosen due to the unequal variances and unequal sample sizes between the COVID-pos and COVID-neg groups. One COVID-pos patient Nfl value of 14,555 pg/ml was considered an outlier and not included in the statistical analysis. Comparison of log Nfl levels across subgroups were analyzed with Kruskal-Wallis ANOVA with p < .05 considered significant. The Kruskal-Wallis ANOVA was chosen due to the non-normal distribution of Nfl levels across groups. Associations between log Nfl levels and other co-morbidities and clinical laboratory variables were analyzed using Pearson correlation and a p value < .05 indicated statistical significance. Linear regression was used to generate a best-fit line. Data were analyzed using GraphPad Prism 8.0.

### 2.3 Human subject protection and protocol approvals

This study and use of patient samples was approved by the University of Arizona Institutional Review Board (IRB# 1410545697). Participant informed consent was provided either directly by the patient or, if the participant was incapacitated, from the patient’s legally authorized representative.

## 3. Results

### 3.1 Demographics

All ICU patients had either an rt-PCR confirmed positive or negative result for SARS-CoV-2 infection. The three groups analyzed included 89 COVID-pos ICU patients, 11 COVID-neg ICU patients, and 8 healthy controls. Both ICU patient groups were recruited from Tucson, AZ between April- August 2020. The healthy adult participants were recruited independently from emergency care personnel in the hospital. Age deciles for each group are detailed in Table 1. The average age of the COVID-pos ICU patients was 60.8, the average age of the COVID-neg ICU patients was 65.1, and the average age of the healthy controls was 51.7.

Demographic and initial admission clinical data for both COVID-pos and COVID-neg ICU patients are detailed in Table 2. Of the 89 COVID-pos ICU patients, we had full demographic and clinical data on 50 patients. All 50 of the COVID-19 positive (COVID-pos) ICU patients were mechanically ventilated. Of these 50 COVID-pos patients, 39 (43.8 percent) did not survive. Of the 11 COVID-neg ICU patients who we had plasma samples from, full demographic and clinical data were available in 5 patients. Of these five patients, four were mechanically ventilated and one was on high flow nasal cannula system. 4 out of the 11 (36.3 percent) did not survive. Levels of critical illness as measured by the total sequential organ failure assessment score (SOFA) were not different between the COVID-pos and COVID-neg groups (9.5±0.4 vs 10.8±2.2, respectively). Cardiovascular disease (CVD) was noted in 35 of the 50 COVID-pos patients and some exhibited more than one type of CVD. This included 29 with hypertension (defined as 140–159/90–99 mm Hg), 10 with hyperlipidemia, 10 with a previous/old MI, 6 with coronary artery disease, 4 with arrhythmia, 2 with heart failure, 1 with valve disease, 3 with obesity and 6 with a smoking history.

Neurological complications (neurocx) were assessed upon hospital admission and during ICU stay. Of the 50 COVID-pos patients, 5 were diagnosed while in the ICU with acute encephalopathy, 5 with ICU delirium, 4 with sleep apnea, 2 with seizure, 5 with depression, one with cerebral edema, 2 with stroke, and one with facial droop. None of the 5 COVID-neg ICU patients had any noted neurological compli-cations.

### 3.2 Nfl Biomarker

**Figure 1:**
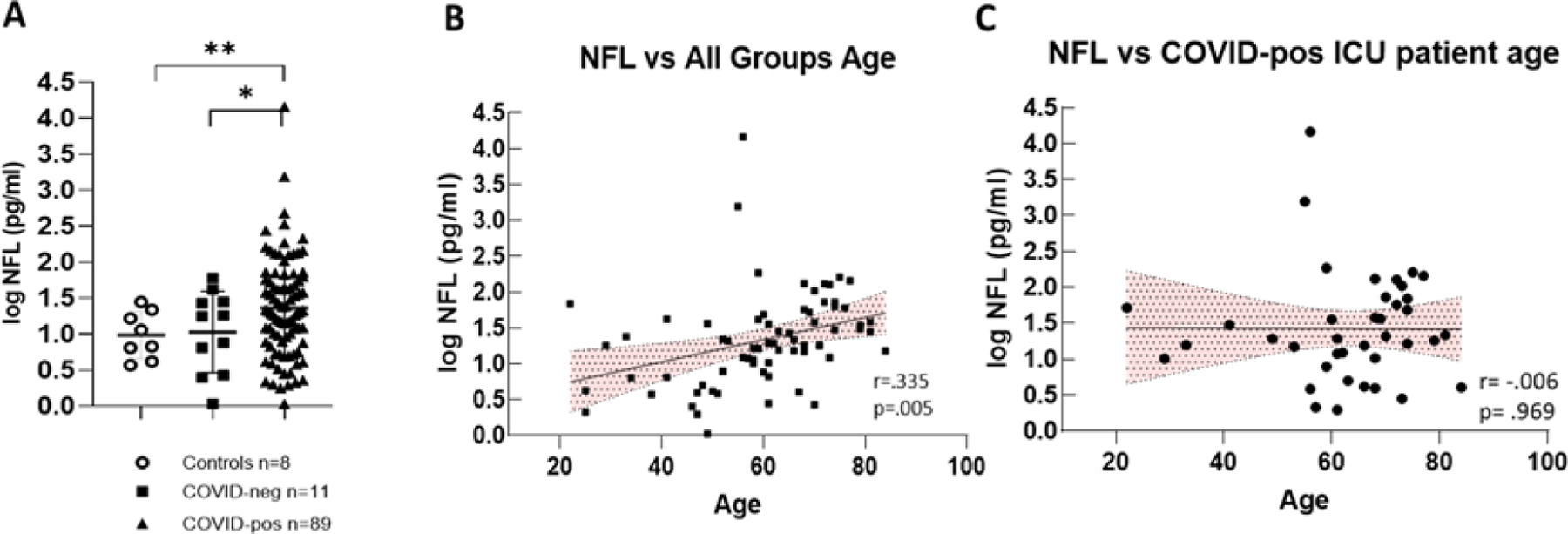
(A) Illustrates log Nfl values from COVID-pos, COVID-neg ICU patients and healthy controls. COVID-pos mean plasma Nfl levels were 66.85±18.9 sem pg/ml, median of 19.8 pg/ml (max 1555-min 1.0 vs. COVID-neg, mean plasma Nfl levels were 19.3±5.6 sem pg/ml, median of 17.8 pg/ml (max 60.2-min 1.0), (95% CI 8.18 to 86.78, two-tailed Welch’s t-test, p = ,01). The 8 healthy controls had mean Nfl levels of 12.3±`3.1 sem pg/ml, median 9.0 pg/ml (max 27.9-min 3.7) and were significantly different from COVID-pos (95% CI 16.27 to 92.71, two-tailed Welches’t-test, p =.005). (B) Log Nfl levels and age from all the groups analyzed together show that log Nfl levels were significantly correlated to age in the combined groups, (r = .335, p =.005, Pearson). (C) COVID-pos ICU patient Nfl levels and age were analyzed separately and there was no significant correlation to age (r = .006, p = .969, Pearson).

In COVID-pos patients in the ICU, Nfl was significantly higher than those observed in COVID-neg ICU patients and healthy control (Figure 1A). COVID-pos patients had a mean plasma Nfl of 229.6±163.9 sem pg/ml, median of 20.4 pg/ml (min 1.0 - max 14,555) vs. COVID-neg patients with a mean plasma Nfl level of 19.3±5.6 sem pg/ml, median of 17.8 pg/ml (min 1.0 - max 60.2), (95% CI 8.18 to 86.78, two-tailed Welch’s t-test, p = .01). The 8 healthy controls had mean Nfl value of 12.3±3.1 sem pg/ml, median 9.0 pg/ml (min 3.7- max 27.9) and were significantly different from COVID-pos (95% CI 16.27 to 92.71, two-tailed Welches’ t-test, p =.005).

To determine if the levels of Nfl in groups were related to subject age, we first performed Pearson correlation analysis of all log Nfl values compared to all group ages (Figure 1B). In this case, the levels of Nfl were significantly positively correlated with age, (r = .335, p =.005, Pearson), as has been previously reported [12, 13]. However, when we performed correlation analysis of Nfl values compared to age in COVID-pos ICU patients alone (Figure 1C), there was no significant correlation of Nfl levels with the age of the COVID-pos patient (r = .006, p = .96).

### 3.3 Nfl and cardiovascular disease

**Figure 2:**
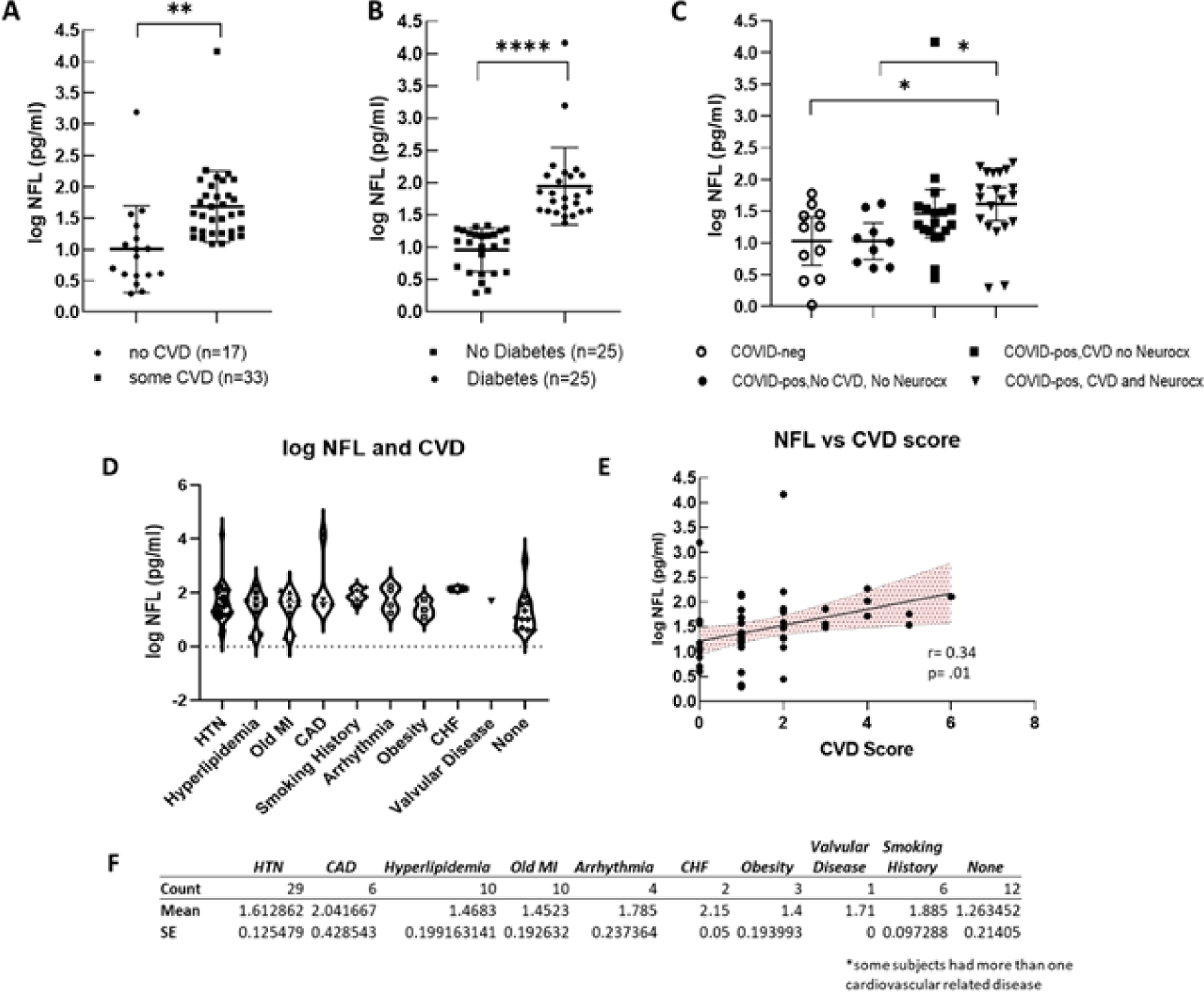
(A) Compares log Nfl values from COVID-pos patients with either some level of CVD or none. The log Nfl levels were significantly higher in COVID-pos patients with some level of CVD (median 1.57, min 1.0-max 4.1 log pg/ml) compared to those with no known CVD (median 0.89, min 0.2 – max 3.1 log pg/ml), (95% CI .27 to 1.0, two-tailed, Welches’s t-test, p = .001). Nfl levels were significantly higher in COVID-pos patients with diabetes (median 1.8 min 1.3 – max 4.1) as compared to those that did not have diabetes (median 1.0, min 0.3 – max 1.3) (95% CI 0.70 to 1.2, two-tailed, Welch’s t-test, p<.0001). (C) Compares log Nfl levels between COVID-neg (n=11), COVID-pos with no CVD and no Neurocx (n=9), COVID-pos with CVD and no Neurocx and COVID-pos (n=18) with both CVD and Neurocx (n=23). While the highest levels of Nfl were seen in COVID-pos with both CVD and Neurocx there was no significant difference in log Nfl levels in COVID-pos with CVD and no Neurocx and those with both. (D) Illustrates the distribution and variance of cardiovascular disease in COVID-pos patients plotted against the level of Nfl for each cardiovascular disease category. (E) Shows the correlation between the levels of Nfl and the CVD score in COVID-pos patients. The levels of NfL were significantly positively correlated with the level of the CVD score (r = 0.34, p = .001, Pearson). (F) Is a table comparing the mean+ sem of log Nfl levels in each of the categories of cardiovascular disease.

There is a clear and well established association between the presence of CVD and the risk of cognitive impairment, neurodegenerative disease and vascular dementia [25–32]. In the present study, we tested the hypothesis that levels of Nfl in COVID-pos ICU patients would be associated with the presence of CVD and diabetes. As seen in Figure 2F, of the 50 COVID-pos patients, 38 had at least one form of CVD with hypertension being the most prevalent with 29/50 COVID-pos patients having a diagnosis of hypertension.

Figure 2D illustrates the distribution and variance of CVD plotted against the level of Nfl for each cardiovascular disease category. As seen in Figure 2A, log Nfl levels were significantly higher in COVID-pos patients with CVD (mean 1.68±.09 sem, median 1.57, min 1.0-max 4.1 log pg/ml) compared to those with no known CVD (mean 1.0±0.16 sem, median 0.89, min 0.2 – max 3.1 log pg/ml), (95% CI .27 to 1.0, two-tailed, Welches’s t-test, p = .001). Likewise, the levels of Nfl in COVID-pos patients were significantly higher in those who had diabetes (mean 1.9±0.1, median 1.8 min 1.3 – max 4.1) as compared to those that did not have diabetes (mean 0.9±.06 sem, median 1.0, min 0.3 – max 1.3) (95% CI 0.70 to 1.2, two-tailed, Welch’s t-test, p<.0001). (Figure 2B).

Figure 2C compares mean log Nfl levels between COVID-neg patients (n=11), COVID-pos patients with no CVD and no neurocx (n=9), COVID-pos with CVD and no neurocx (n=18) and those with both CVD and neurocx (n=23). While the highest levels of Nfl were seen in COVID-pos patients with both CVD and neurocx there was no significant difference in log Nfl levels in COVID-pos with CVD and no neurocx and those with both. The log Nfl levels in COVID-pos patients were positively correlated with the composite CVD Score (Figure 2E), (r = 0.34, p = .001, Pearson) suggesting that the Nfl levels in these COVID-pos patients are related to the level of CVD.

### 3.4 Neurofilament light and neurological complications

**Figure 3:**
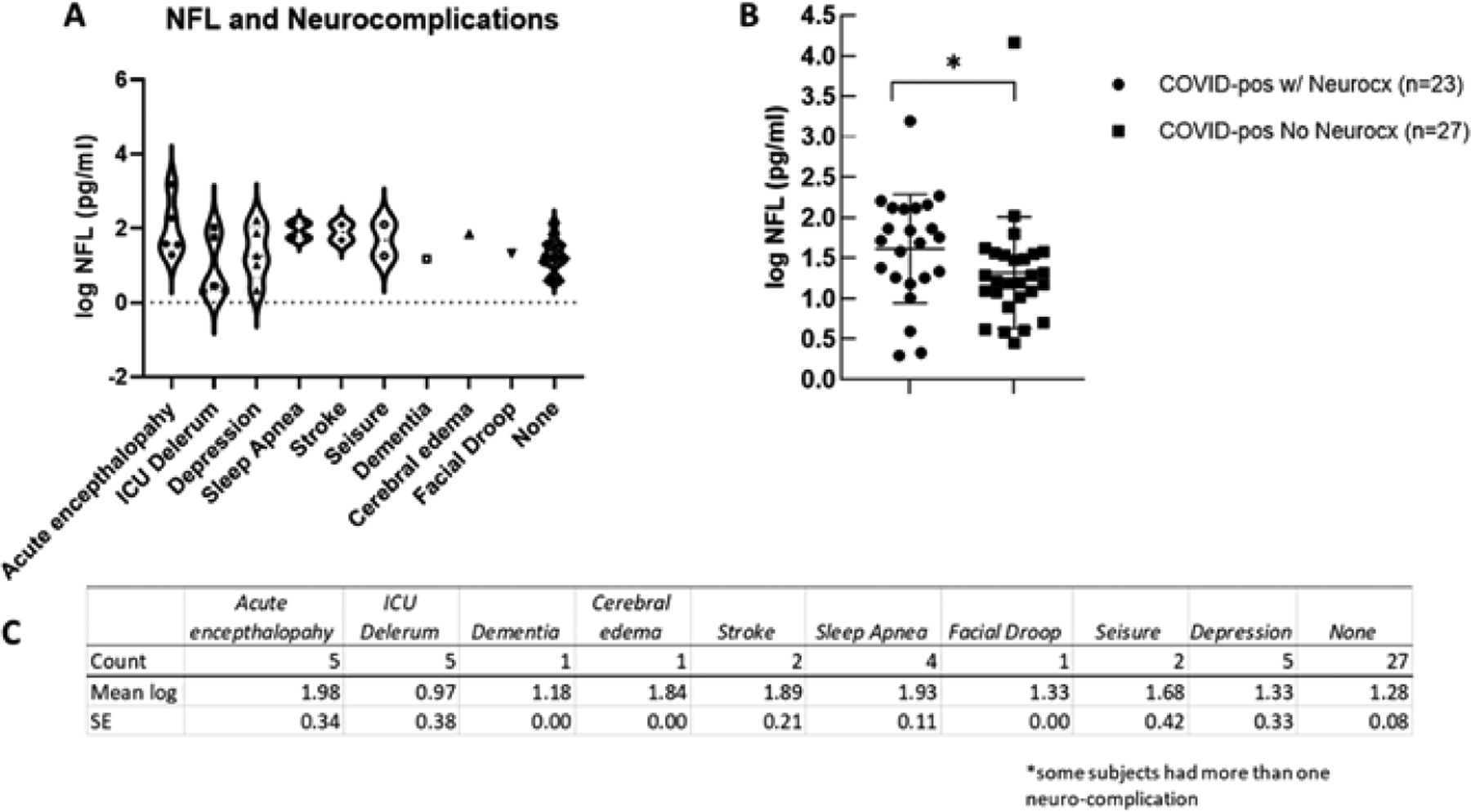
(A) Illustrates the distribution and variance of neurological complications in COVID-pos patients plotted against the level of log Nfl for each neurological complication category. (B) Illustrates a significant difference of log Nfl levels in COVID-pos patients with neurological complications (1.6±0.1, median 1.7, min 0.29- max 3.1, n=23) to COVID-pos patients with no observed neurological complications (1.2±.07, median 1.2, min 0.4 – max 2.0, n=27), 95% CI 0.7 to .73, two-tailed Welch’s t-test, p =.01). (C) Is a table comparing the mean ± sem of log Nfl levels in each of the categories of neurological complication.

To determine if the levels of Nfl were related to the presence of neurocx, we compared Nfl levels in COVID-pos ICU patients in those that had at least one reported neurocx to those that had none. Of the 50 COVID-pos ICU patients, 23 exhibited at least one or more neurocx and 27 had no neurocx. Of these 23, all also had at least one or more noted diagnosis of CVD. The types and distributions of neurocx in the COVID-pos patients are seen in Figures 3A and 3C. None of the 5 COVID-neg ICU patients exhibited any neurocx. Figure 3B illustrates significant differences in mean log Nfl levels in COVID-pos with neurocx (mean 1.6±0.1, median 1.7, min 0.29 - max 3.1, n=23) compared to COVID-pos with no observed neurocx (mean 1.2±.07, median 1.2, min 0.4 – max 2.0, n=27), 95% CI 0.7 to .73, two-tailed Welch’s t-test, p =.01).

### 3.5 Nfl Levels: ICU status and clinical blood panel markers

**Figure 4:**
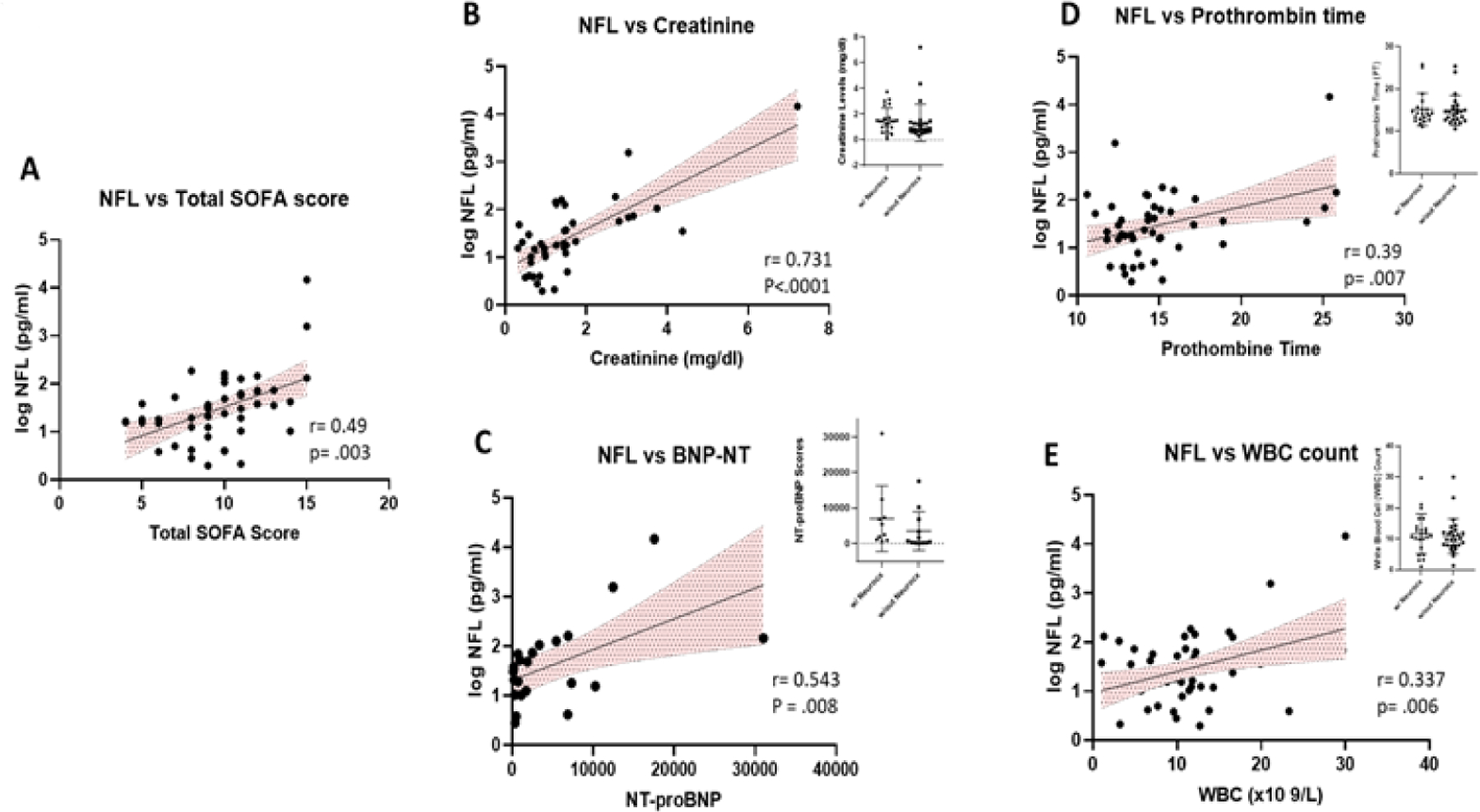
(A) Illustrates the positive correlation between the levels of Nfl and the total SOFA score in COVID-pos patients (r = 0.49, p =.003, Pearson). In addition, there was a positive correlation in COVID-pos patients between the levels of Nfl and (B) creatinine, r =.73, p<.0001, Pearson), (C) BNP-NT, r=0.54, p =.008, Pearson (D) prothrombin time, r=0.38, p = .007, Pearson and (E) WBC count, r = 0.337, p = .006, Pearson.

All 50 of the COVID-pos patients exhibited sepsis and multiple organ system failure and systemic inflammatory response syndrome (SIRS), all of which have been associated with post ICU cognitive impairment [24, 33]. We evaluated the association between COVID-pos Nfl levels and the ICU clinical status and sequential organ failure assessment core (SOFA). As seen in Figure 4A, in COVID-pos patients, the levels of Nfl were correlated with the ICU SOFA score suggesting that the levels of Nfl are related to the level of systemic inflammation and organ failure in COVID-pos ICU patients (r = 0.49, p = .003, Pearson). We also performed a correlation analysis of Nfl and results from 33 different clinical blood panel markers. Results from the correlations that were significant (p<.05) are illustrated in Figures 4B–4E. The log Nfl values were significantly correlated with levels of (B) creatinine (r = 0.7, p<.0001, Pearson), (C) N terminal pro B type natriuretic peptide (BNP-NT, r = 0.54, p = .008, Pearson), (D) prothrombin time (PT) (r = .39, p = .007, Pearson) and (E) the white blood cell count (WBC, r = 0.33, p =.006, Pearson). The inserts for each figure show that there are no differences in the levels of each of the blood markers in Figures 4B–4E between COVID-pos patients with and without neurological complication suggesting the relationship between log Nfl levels and levels of blood creatinine, BNP-NT, PT and WBC were not related to the presence or absence of neurological complications.

## 4. Discussion

In our cohort of 100 patients hospitalized in the ICU in Tucson, Arizona between April 2020 and August 2020, 89 tested positive for COVID-19 and 11 tested negative. We measured serum levels of Nfl in all of these patients and compared them to each other and a cohort of healthy controls (n=8). The mean levels of Nfl in the COVID-pos patients were significantly higher as compared to those observed in COVID-neg patients and healthy controls and suggest that COVID-pos ICU patients may have CNS injury. Of the 50 COVID-pos patients from which we had clinical data, 70% also had at least one or more diagnosis of one of the subtypes of CVD that included heart failure, hypertension (defined as > 140–159/90–99 mm Hg), coronary artery disease, valvular disease, arrhythmia, hyperlipidemia, obesity, and smoking history. The levels of Nfl in these patients with CVD were 205% higher than in those COVID-pos patients those with no CVD. Likewise, those COVID-pos patients with diabetes had 102% higher levels of Nfl than those without diabetes. The levels of Nfl were similar in COVID-pos patients with CVD alone and no neurocx as compared to COVID-pos patients with both CVD and neurocx, suggesting that the increased levels of Nfl in COVID-pos patients with CVD was not dependent on the presence or absence of neurocx.

Numerous studies have shown that Nfl levels in both the serum and CSF have been validated to be able to detect brain injury and axonal damage in individuals with neurodegenerative diseases [12–14], brain trauma [19] as well as cardiovascular disease and cardiac surgery [17, 18]. A number of studies have shown that cognitive impairment and neurodegenerative disease are correlated with vascular disease, inflammation and decreased cerebral brain blood flow [25–30]. Mechanisms thought to contribute to cognitive impairment in patients with chronic CVD and diabetes includes high levels of systemic inflammation [34], altered cerebrovascular autoregulation [35], and microembolism [36]. Inflammatory processes play an important role in CVD-related increases in circulating inflammatory mediators are seen in the brain.

An early study of 214 COVID-19 patients in China was among the first to report evidence of neurological complications of SARS-CoV2 infection [5]. The symptoms in these patients were categorized into three areas including changes to 1) the central nervous system (CNS) which included patient reports of dizziness, headache, ataxia, impaired consciousness, and acute cerebrovascular disease; 2) the peripheral nervous system (PNS) symptoms (loss of smell, taste and some loss of peripheral sensation); and 3) skeletal muscular dysfunction. In these studies, 41.1% of the subjects exhibited severe COVID-19 disease requiring ventilation. In these patients, 36.3% also had hypertension and 45.5% had neurological manifestations. It has been suggested that the CNS complications in COVID-19 may be due ACE2 expression in the CNS and the PNS [37]. Given that ACE2 is known to be expressed in brain endothelial cells within the cerebrovascular system and on glia and neurons [38], it has been suggested that SARS-Cov-2 binding to ACE2 in brain vascular endothelium may result in a compromised blood-brain-barrier (BBB). This compromise would allow entry of the virus into the brain parenchyma leading to virus induced neuronal inflammation and damage [39, 40].

We also found a significant positive correlation of Nfl levels with ICU clinical status and the SOFA score and four blood chemistry measurements including creatinine, prothrombin time (PT), WBC counts and NT-pro-BNP. Alterations in coagulation disorders have recently been reported in other COVID-19 studies [41–45] and have been suggested to be related to microvascular disease, capillary leakage and poor prognosis in COVID-19 patients. Increases in NT-pro-BNP is a well-known clinical biomarker for heart disease and has also been shown to be affiliated with microvascular disease in the brain, kidney and heart [46] and cardiac complications and mortality rates in COVID-19 patients [47, 48].

Taken together, our data suggest that increased levels of Nfl in COVID-19 ICU patients are related to not only to the neurological complications seen in these patients, but also to the presence and extent of chronic inflammatory diseases such as cardiovascular disease and diabetes. In older adults, chronic systemic inflammatory-related diseases, such as vascular disease, heart failure and hypertension that lead to increased brain and systemic inflammation and decreased brain perfusion, are known to increase the risk for dementia and the development of VCID [49–52]. The COVID-19 related cytokine storm and high levels of proinflammatory cytokines along with hypoxia due to respiratory dysfunction and concomitant CVD seen in COVID-19 ICU patients are likely to result in short and long-term cognitive dysfunction and may accelerate pre-existing cognitive deficits [53, 54, 40].

## 5. Limitations

This study has several limitations. First, there are a limited number of subjects in all groups and an even a further limitation on the numbers of subjects we had access to full clinical data. We were not able to obtain consent from all ICU participants who significantly limited our ability to perform full clinical data comparisons between the COVID-pos and COVID-neg subjects.

Also, our healthy control sample, while similar in age-range, was small and included no baseline clinical comorbidity data. Future studies with larger cohorts and complete clinical records will be needed to fully understand the relationship between Nfl and ICU patient status. Lastly, we have no uniform neurological or cognitive assessments in the ICU patients which make it impossible to correlate these Nfl levels to cognitive function.

## 6. Conclusions

Increased levels of Nfl in COVID-19 ICU patients are correlated with both neurological complications and with the presence of cardiovascular disease and diabetes. Nfl may serve as a biomarker for the risk of cognitive and neurological impairment in COVID-19 patients admitted to the ICU.

## Figures and Tables

**Table 1:** Age deciles of cohort groups.

Age Decile									
Participant Cohort	n	Avg Age	20–29	30–39	40–49	50–59	60–69	70–79	80–89
COVID-19-pos ICU patients	89	60.8	3	1	5	11	15	13	2
COVID-19-neg ICU patients	11	65.1	-	-	1	1	-	4	-
Control healthy adults	8	51.7	1	1	1	3	1	-	1

**Table 2:** Demographic, clinical and Nfl levels.

Group Characteristics	COVID-pos	COVID-neg	Controls
Total n Nfl data (Nfl with clinical data)	89 (50)	11 (5)	8
Sex, n (%)			
Female	26 (52)	5	4
Male	24 (48)	6	4
Ethnicity-Hispanic origin, n,(%)	26 (52)	2 (18)	-
Ethnicity -non-Hispanic origin, n (%)	20 (40)	9 (82)	-
Ethnicity unknown, n (%)	4 (8)	0	-
Primary diagnosis of Acute Hypoxic Respiratory Failure n, (%)	50 (100)	4 (66)	-
Secondary Diagnosis - Cardiovascular disease/hypertension n, (%)	35 (70)	4 (66)	-
Secondary Diagnosis - Neurological complications n (%)	2 (46)	0	-
Diabetes, n (%)	25 (50)	2 (33)	-
BMI	34.1±1	28.5±1.6	-
Smoking History, n (%)	8 (16)	1 (16)	-
Nfl (pg/ml,mean±sem, median, max,min)	229.6±163, 20.4 (1–14,559)	19.3±15.6, 17.8 (1–60)	12.3±13.1, 9.0 (4–28)

## Data Availability

Researchers can request for access to anonymized data from the present study for well-defined research questions that are consistent with the overall research agenda for the cohort. Please contact the corresponding author.

## References

[1] Dolatshahi M, Sabahi M, Aarabi MH. Pathophysiological Clues to How the Emergent SARS-CoV-2 Can Potentially Increase the Susceptibility to Neurodegeneration. Mol Neurobiol (2021). 10.1007/s12035-020-02236-2 PMC7791539 33417221

[2] Ghaffari M, Ansari H, Beladimoghadam N, Aghamiri SH, Haghighi M, Neurological features and outcome in COVID-19: dementia can predict severe disease. J Neurovirol (2021). 10.1007/s13365-020-00918-0 PMC7792552 33417193

[3] Karimi L, Sales C, Crewther SG, Wijeratne T. Acute Ischemic Stroke in SARS-CoV, MERS-CoV, SARS-CoV-2: Neurorehabilitation Implications of Inflammation Induced Immunological Responses Affecting Vascular Systems. Front Neurol 11 (2020): 565665. 33414753 10.3389/fneur.2020.565665 PMC7783449

[4] Helms J, Kremer S, Merdji H, Clere-Jehl R, Schenck M, Neurologic Features in Severe SARS-CoV-2 Infection. N Engl J Med 382 (2020): 2268–2270. 32294339 10.1056/NEJMc2008597 PMC7179967

[5] Mao L, Wang M, Chen S, He Q, Chang J, Neurological Manifestations of HospitalizedPatients with COVID-19 in Wuhan, China: a retrospective case series. medRxiv (2020).

[6] Brown EE, Kumar S, Rajji TK, Pollock BG, Mulsant BH. Anticipating and Mitigating the Impact of the COVID-19 Pandemic on Alzheimer’s disease and Related Dementias. Am J Geriatr Psychiatry 28 (2020): 712–721. 32331845 10.1016/j.jagp.2020.04.010 PMC7165101

[7] Varatharaj A, Thomas N, Ellul MA, Davies NWS, Pollak TA, Neurological and neuropsychiatric complications of COVID-19 in 153 patients: a UK-wide surveillance study. Lancet Psychiatry 7 (2020): 875–882. 32593341 10.1016/S2215-0366(20)30287-X PMC7316461

[8] Paterson RW, Brown RL, Benjamin L, Nortley R, Wiethoff S, The emerging spectrum of COVID-19 neurology: clinical, radiological and laboratory findings. Brain (2020). 10.1093/brain/awaa240 PMC7454352 32637987

[9] Guan WJ, Ni ZY, Hu Y, Liang WH, Ou CQ, Clinical Characteristics of Coronavirus Disease 2019 in China. N Engl J Med (2020). 10.1056/NEJMoa2002032 PMC7092819 32109013

[10] Sardu C, Gambardella J, Morelli MB, Wang X, Marfella R, Hypertension, Thrombosis, Kidney Failure, and Diabetes: Is COVID-19 an Endothelial Disease? A Comprehensive Evaluation of Clinical and Basic Evidence. J Clin Med 9 (2020). 10.3390/jcm9051417 PMC7290769 32403217

[11] Zetterberg H Neurofilament Light: A Dynamic Cross-Disease Fluid Biomarker for Neurodegeneration. Neuron 91 (2016): 1–3. 27387643 10.1016/j.neuron.2016.06.030

[12] Khalil M, Teunissen CE, Otto M, Piehl F, Sormani MP, Neurofilaments as biomarkers in neurological disorders. Nat Rev Neurol 14 (2018): 577–589. 30171200 10.1038/s41582-018-0058-z

[13] Quiroz YT, Zetterberg H, Reiman EM, Chen Y, Su Y, Plasma neurofilament light chain in the presenilin 1 E280A autosomal dominant Alzheimer’s disease kindred: a cross-sectional and longitudinal cohort study. Lancet Neurol 19 (2020): 513–521. 32470423 10.1016/S1474-4422(20)30137-X PMC7417082

[14] Shahim P, Politis A, van der Merwe A, Moore B, Chou YY, Neurofilament light as a biomarker in traumatic brain injury. Neurology 95 (2020): e610–e622. 32641538 10.1212/WNL.0000000000009983 PMC7455357

[15] Zetterberg H Is There a Value of Neurofilament Light as a Biomarker for Neurodegeneration in Parkinson’s disease? Mov Disord 35 (2020): 1111–1112. 32691913 10.1002/mds.28101

[16] Nielsen HH, Soares CB, Høgedal SS, Madsen JS, Hansen RB, Acute Neurofilament Light Chain Plasma Levels Correlate with Stroke Severity and Clinical Outcome in Ischemic Stroke Patients. Front Neurol 11 (2020): 448. 32595585 10.3389/fneur.2020.00448 PMC7300211

[17] Wiberg S, Holmgaard F, Blennow K, Nilsson JC, Kjaergaard J, Associations between mean arterial pressure during cardiopulmonary bypass and biomarkers of cerebral injury in patients undergoing cardiac surgery: secondary results from a randomized controlled trial. Interact Cardiovasc Thorac Surg (2020). 10.1093/icvts/ivaa264 PMC8906782 33221914

[18] Polymeris AA, Coslovksy M, Aeschbacher S, Sinnecker T, Benkert P, Serum neurofilament light in atrial fibrillation: clinical, neuroimaging and cognitive correlates. Brain Commun 2 (2020): fcaa166. 33381755 10.1093/braincomms/fcaa166 PMC7753055

[19] Thelin E, Al Nimer F, Frostell A, Zetterberg H, Blennow K, A Serum Protein Biomarker Panel Improves Outcome Prediction in Human Traumatic Brain Injury. J Neurotrauma 36 (2019): 2850–2862. 31072225 10.1089/neu.2019.6375 PMC6761606

[20] Kanberg N, Ashton NJ, Andersson LM, Yilmaz A, Lindh M, Neurochemical evidence of astrocytic and neuronal injury commonly found in COVID-19. Neurology (2020). 10.1212/WNL.0000000000010111 32546655

[21] Sutter R, Hert L, De Marchis GM, Twerenbold R, Kappos L, Serum neurofilament light chain levels in the intensive care unit: comparison between severely ill patients with and without COVID-19. Ann Neurol (2020). 10.1002/ana.26004 33377539

[22] Edén A, Kanberg N, Gostner J, Fuchs D, Hagberg L, CSF biomarkers in patients with COVID-19 and neurological symptoms: A case series. Neurology (2020). 10.1212/WNL.0000000000010977 33004602

[23] de Grooth HJ, Geenen IL, Girbes AR, Vincent JL, Parienti JJ, Oudemans-van Straaten HM. SOFA and mortality endpoints in randomized controlled trials: a systematic review and meta-regression analysis. Crit Care 21 (2017): 38. 28231816 10.1186/s13054-017-1609-1 PMC5324238

[24] Ely EW, Margolin R, Francis J, May L, Truman B, Evaluation of delirium in critically ill patients: validation of the Confusion Assessment Method for the Intensive Care Unit (CAM-ICU). Crit Care Med 29 (2001): 1370–1379. 11445689 10.1097/00003246-200107000-00012

[25] Kapasi A, Schneider JA. Vascular contributions to cognitive impairment, clinical Alzheimer’s disease, and dementia in older persons. Biochim Biophys Acta 1862 (2016): 878–886. 10.1016/j.bbadis.2015.12.023 PMC11062590 26769363

[26] Toledo JB, Toledo E, Weiner MW, Jack CR, Jagust W, Cardiovascular risk factors, cortisol, and amyloid-β deposition in Alzheimer’s Disease Neuroimaging Initiative. Alzheimers Dement 8 (2012): 483–489. 23102118 10.1016/j.jalz.2011.08.008 PMC3668456

[27] Yarchoan M, Xie SX, Kling MA, Toledo JB, Wolk DA, Cerebrovascular atherosclerosis correlates with Alzheimer pathology in neurodegenerative dementias. Brain 135 (2012): 3749–3756. 23204143 10.1093/brain/aws271 PMC3577102

[28] van Oijen M, de Jong FJ, Witteman JC, Hofman A, Koudstaal PJ, Atherosclerosis and risk for dementia. Ann Neurol 61 (2007): 403–410. 17328068 10.1002/ana.21073

[29] Gorelick PB, Furie KL, Iadecola C, Smith EE, Waddy SP, Defining Optimal Brain Health in Adults: A Presidential Advisory from the American Heart Association/American Stroke Association. Stroke 48 (2017): e284–e303. 28883125 10.1161/STR.0000000000000148 PMC5654545

[30] Santos CY, Snyder PJ, Wu WC, Zhang M, Echeverria A, Pathophysiologic relationship between Alzheimer’s disease, cerebrovascular disease, and cardiovascular risk: A review and synthesis. Alzheimers Dement (Amst) 7 (2017): 69–87. 28275702 10.1016/j.dadm.2017.01.005 PMC5328683

[31] Canobbio I, Abubaker AA, Visconte C, Torti M, Pula G. Role of amyloid peptides in vascular dysfunction and platelet dysregulation in Alzheimer’s disease. Front Cell Neurosci 9 (2015): 65. 25784858 10.3389/fncel.2015.00065 PMC4347625

[32] Janota C, Lemere CA, Brito MA. Dissecting the Contribution of Vascular Alterations and Aging to Alzheimer’s disease. Mol Neurobiol 53 (2016): 3793–3811. 26143259 10.1007/s12035-015-9319-7

[33] Brummel NE, Hughes CG, Thompson JL, Jackson JC, Pandharipande P, Inflammation and Coagulation during Critical Illness and Long-Term Cognitive Impairment and Disability. Am J Respir Crit Care Med (2020). 10.1164/rccm.201912-2449OC PMC7958515 33030981

[34] Athilingam P, Moynihan J, Chen L, D’Aoust R, Groer M, Elevated levels of interleukin 6 and C-reactive protein associated with cognitive impairment in heart failure. Congest Heart Fail 19 (2013): 92–98. 23057677 10.1111/chf.12007 PMC3801169

[35] Zuccalà G, Onder G, Pedone C, Cocchi A, Carosella L, Cognitive dysfunction as a major determinant of disability in patients with heart failure: results from a multicentre survey. On behalf of the GIFA (SIGG-ONLUS) Investigators. J Neurol Neurosurg Psychiatry 70 (2001): 109–112. 11118258 10.1136/jnnp.70.1.109 PMC1763482

[36] Almeida OP, Garrido GJ, Beer C, Lautenschlager NT, Arnolda L, Cognitive and brain changes associated with ischaemic heart disease and heart failure. Eur Heart J 33 (2012):1769–1776. 22296945 10.1093/eurheartj/ehr467

[37] Bader M ACE2, angiotensin-(1–7), and Mas: the other side of the coin. Pflugers Arch 465 (2013):79–85. 23463883 10.1007/s00424-012-1120-0

[38] Turner AJ, Hiscox JA, Hooper NM. ACE2: from vasopeptidase to SARS virus receptor. Trends Pharmacol Sci 25 (2004): 291–294. 15165741 10.1016/j.tips.2004.04.001 PMC7119032

[39] Baig AM, Khaleeq A, Ali U, Syeda H. Evidence of the COVID-19 Virus Targeting the CNS: Tissue Distribution, Host-Virus Interaction, and Proposed Neurotropic Mechanisms. ACS Chem Neurosci 11 (2020): 995–998. 32167747 10.1021/acschemneuro.0c00122

[40] Ahmed MU, Hanif M, Ali MJ, Haider MA, Kherani D, Neurological Manifestations of COVID-19 (SARS-CoV-2): A Review. Front Neurol 11 (2020): 518. 32574248 10.3389/fneur.2020.00518 PMC7257377

[41] Calderon-Lopez MT, Garcia-Leon N, Gomez-Arevalillo S, Martin-Serrano P, Matilla-Garcia A. Coronavirus disease 2019 and coagulopathy: other prothrombotic coagulation factors. Blood Coagul Fibrinolysis 32 (2021): 44–49. 10.1097/MBC.0000000000000996 33417336

[42] Rahi MS, Jindal V, Reyes SP, Gunasekaran K, Gupta R, Hematologic disorders associated with COVID-19: a review. Ann Hematol (2021). 10.1007/s00277-020-04366-y PMC7789889 33415422

[43] Robba C, Battaglini D, Ball L, Valbusa A, Porto I, Coagulative Disorders in Critically Ill COVID-19 Patients with Acute Distress Respiratory Syndrome: A Critical Review. J Clin Med 10 (2021). 10.3390/jcm10010140 PMC7795033 33401632

[44] Yuan X, Tong X, Wang Y, Wang H, Wang L, Coagulopathy in elderly patients with coronavirus disease 2019. Aging Med (Milton) 3 (2020): 260–265. 10.1002/agm2.12133 PMC7771561 33392432

[45] Long H, Nie L, Xiang X, Li H, Zhang X, D-Dimer and Prothrombin Time Are the Significant Indicators of Severe COVID-19 and Poor Prognosis. Biomed Res Int 2020 (2020): 6159720. 32596339 10.1155/2020/6159720 PMC7301188

[46] Nowroozpoor A, Gutterman D, Safdar B. Is microvascular dysfunction a systemic disorder with common biomarkers found in the heart, brain, and kidneys? - A scoping review. Microvasc Res 134 (2020): 104123. 33333140 10.1016/j.mvr.2020.104123

[47] Bansal A, Kumar A, Patel D, Puri R, Kalra A, Meta-analysis Comparing Outcomes in Patients With and Without Cardiac Injury and Coronavirus Disease 2019 (COVID 19). Am J Cardiol (2020). 10.1016/j.amjcard.2020.11.009 PMC7671934 33217345

[48] Rath D, Petersen-Uribe Á, Avdiu A, Witzel K, Jaeger P, Impaired cardiac function is associated with mortality in patients with acute COVID-19 infection. Clin Res Cardiol 109 (2020): 1491–1499. 32537662 10.1007/s00392-020-01683-0 PMC7293880

[49] Suzuki H, Matsumoto Y, Ota H, Sugimura K, Takahashi J, Hippocampal Blood Flow Abnormality Associated With Depressive Symptoms and Cognitive Impairment in Patients With Chronic Heart Failure. Circ J 80 (2016): 1773–1780. 27295999 10.1253/circj.CJ-16-0367

[50] Love S, Miners JS. Cerebral Hypoperfusion and the Energy Deficit in Alzheimer’s disease. Brain Pathol 26 (2016): 607–617. 27327656 10.1111/bpa.12401 PMC8028913

[51] Miners JS, Schulz I, Love S. Differing associations between Aβ accumulation, hypoperfusion, blood-brain barrier dysfunction and loss of PDGFRB pericyte marker in the precuneus and parietal white matter in Alzheimer’s disease. J Cereb Blood Flow Metab (2017): 271678X17690761. 10.1177/0271678X17690761 PMC5757436 28151041

[52] Cermakova P, Eriksdotter M, Lund LH, Winblad B, Religa P, Heart failure and Alzheimer’s disease. J Intern Med 277 (2015): 406–425. 25041352 10.1111/joim.12287 PMC4409079

[53] Heneka MT, Golenbock D, Latz E, Morgan D, Brown R. Immediate and long-term consequences of COVID-19 infections for the development of neurological disease. Alzheimers Res Ther 12 (2020): 69. 32498691 10.1186/s13195-020-00640-3 PMC7271826

[54] Aghagoli G, Gallo Marin B, Katchur NJ, Chaves-Sell F, Asaad WF, Neurological Involvement in COVID-19 and Potential Mechanisms: A Review. Neurocrit Care (2020). 10.1007/s12028-020-01049-4 PMC7358290 32661794

